# A Mobile, Avatar-Based App for Improving Body Perceptions Among Adolescents: A Pilot Test

**DOI:** 10.2196/games.6354

**Published:** 2017-03-02

**Authors:** Annmarie A Lyles, Ashish Amresh, Jennifer Huberty, Michael Todd, Rebecca E Lee

**Affiliations:** ^1^ College of Nursing and Health Innovation Arizona State University Phoenix, AZ United States; ^2^ Ira A Fulton School of Engineering Arizona State University Tempe, AZ United States; ^3^ School of Nutrition and Health Promotion Arizona State University Phoenix, AZ United States; ^4^ Center for Health Promotion and Disease Prevention College of Nursing and Health Innovation Arizona State University Phoenix, AZ United States

**Keywords:** adolescents, avatars, eHealth, mHealth, perceptions, Web-based, usability testing

## Abstract

**Background:**

One barrier to effectively treating weight issues among adolescents is that they tend to use social comparison instead of objective measures to evaluate their own health status. When adolescents correctly perceive themselves as overweight, they are more likely to adopt healthy lifestyle behaviors.

**Objective:**

The purpose of this pilot test was to develop and assess acceptability and usability of an avatar-based, theoretically derived mobile app entitled Monitor Your Avatar (MYA).

**Methods:**

The MYA app was engineered for high school adolescents to identify, using avatars, what they thought they looked like, what they wanted to look like, and what they actually looked like based on body measurements.

**Results:**

The MYA app was pilot-tested with male and female adolescents aged 15-18 years to assess for acceptability and usability. A total of 42 students created and viewed their avatars. The majority of the adolescents were female (28/42, 67%), age 16 years (16/42, 38%), white (35/42, 83%), non-Hispanic (36/42, 86%), in grade 10 (20/42, 48%), healthy weight for females (23/28, 82%), and obese for males (7/14, 50%). The adolescents had positive reactions to the avatar app and being able to view avatars that represented them. All but one student (41/42, 98%) indicated some level of comfort viewing the avatars and would use the app in the future to see how their bodies change over time.

**Conclusions:**

Avatar-based mobile apps, such as the MYA app, provide immediate feedback and allow users to engage with images that are personalized to represent their perceptions and actual body images. This pilot study adds to the increasing but limited research of using games to improve health outcomes among high school adolescents. There is a need to further adapt the MYA app and gather feedback from a larger number of high school adolescents, including those from diverse backgrounds.

## Introduction

One in five adolescents in the United States are obese, increasing their risk for related complications such as poor quality of life, asthma, type 2 diabetes, and hypertension. Additionally, as they age, adolescents’ participation in physical activity decreases [1], while concern about weight and negative body image increases [2].

One barrier to treating these adolescent weight issues is that they tend to use social comparison instead of objective measures to evaluate their own health status [3]. Having inaccurate body size perceptions may decrease adolescents’ likelihood of changing their health behaviors [4]. If adolescents correctly perceive themselves as overweight, they are more likely to adopt healthy lifestyle behaviors [5] than will those who do not recognize themselves as overweight [4].

Another barrier to effectively treating weight issues among adolescents is misclassifying obesity risk due to incomplete measures for adolescents. Researchers and health care providers have long used body mass index (BMI), a common, inexpensive measure, to determine healthy weight in adolescents. BMI does not distinguish between fat and muscle, does not identify body size perceptions, and cannot detect those at risk for unnecessary and unsafe weight loss behaviors. There is a potential for adolescents to not receive appropriate counseling and referral if their body fat percentage, perceptions, and methods for losing weight are not assessed along with BMI [6].

Adolescents also contend with using the current methods to assess healthy body sizes. Using BMI may be too vague or abstract for adolescents to comprehend. Their weight in pounds on a scale may change insignificantly or not at all even though positive lifestyle changes are substantial. Choosing from a series of body figure silhouettes may not be true representations of the adolescents’ body shapes. These methods of assessing body weight classifications and changes made to the bodies may prevent adolescents from seeing the effects that healthy eating and physical activity can have on their health.

As children move into adolescence, they are more likely to desire independence in attaining and maintaining healthy bodies. Identification (ie, the extent an individual relates to a model and feels similarity to the model) can increase the likelihood for a teen to perform a learned behavior [3,7]. The power of identification increases when the models are of the same sex [4], race [5], or skill level [6]. Fox and Bailenson [8] conducted three studies and found virtual self-models can be an effective impetus for health behavior change. Virtual self-models such as avatars (ie, computerized representations of the adolescents’ bodies) can be connected to goal setting, self-monitoring, direct reinforcement, and social support processes that drive behavior change. Having adolescents interact with avatars on mobile devices, such as tablet computers, engages them in viewing and thinking about their bodies in a realistic and positive light.

Visually rich 3D representations that accurately portray how bodies appear may have a greater impact on adolescent behavior than can a number on a scale or BMI percentage. In addition, adolescents prefer Internet-based health resources because of the 24-hour availability and lack of perceived judgment and conflict with sensitive topics [9]. Mobile phone technology has the ability to track everyday behavior changes in an unobtrusive way, in real time [10,11], and with the potential to provide immediate feedback [12].

Researchers and providers can have a greater impact on adolescents’ actions to change their bodies or help maintain healthy bodies by providing interactive and dynamic strategies. Further, over 93% of teens are active users of the Internet [13], 75% own mobile phones [14], and 40% own iPhones [15], suggesting that Web- and mobile device-based apps can be powerful technologies for implementing behavior change programs in this population.

The purpose of this pilot test was to develop and assess acceptability and usability of an avatar-based, theoretically derived, mobile app entitled *Monitor Your Avatar* (MYA). Positive lifestyle changes can affect adolescents’ body composition, which in turn can affect their perceptions of their bodies, their body shapes, their satisfaction with their bodies, and their emotional well-being as they mature into adulthood.

## Methods

### Design and Development of the App

The MYA app was engineered for male and female high school adolescents to identify, using avatars, what they thought they looked like, what they wanted to look like, and what they actually looked like based on body measurements. The MYA app is interactive and designed for adolescents to change specific body parts of the avatars; they can also access the app recurrently to help monitor their targeted goals. The completion of the app consisted of three phases: classification, development, and prototype testing. The three phases of the project were derived from combining a model-driven approach to developing software [16] along with an iterative user-centered design approach to creating mobile health apps [17]. The classification phase required the collection of accurate datasets, the development phase developed the models based on this data, and the prototype-testing phases incorporated user testing to validate the models developed in the previous stage. All policies, procedures, and ethical concerns for all phases were approved by the Institutional Review Board of the university and high school. All parents provided informed consent and all adolescents provided assent to participate.

### Classification Based on Body Scans

The purpose of the classification phase was to classify the models based on body scans for use when creating the three avatars.

#### Procedure for Body Scans

In order to develop a classification, it was necessary to body-scan male and female adolescents 15-18 years of age. Regarding recruitment, subject inclusion criteria were as follows: (1) in grades 9-12; (2) able to speak, read, and write English; (3) able to stand on a turntable for the 1-minute body scan; and (4) comfortable wearing form-fitting clothes such as compression shorts, leggings, form-fitting tank top, or swimwear. MyBodee by Styku [15] was used to scan the adolescents. MyBodee [15] is a portable and highly accurate body measurement technology. The adolescents wore form-fitting clothes and stood on a turntable. Using a tablet and 3D camera, the researcher measured each adolescent’s full body shape. The adolescent slowly spun a full 360 degrees on a safe and automated turntable. After 40 seconds, the adolescent’s scan was sent to a secure, private network where the research team concurrently analyzed the shape and body measurements. The research team also collected additional body measurements, including the widths and circumferences of the chest, biceps, waist, hips, thigh, and calf using a body tape measure. The scan did not measure the widths of these body parts, so collection of the circumferences and widths of the body parts was needed to configure ratios to be used during the classification phase. A total of 47 adolescents 15-18 years of age were scanned—24 male and 23 female—using MyBodee [15]. MyBodee [15] was only needed to scan the adolescents so their body scans could be used to classify the models for use in the app.

#### Classification

Before creating the models, the research team classified the scanned models using the ratio of height and weight. According to the ratio, we classified both male and female models into six groups. In each group, we selected one or two of the most representative models and reviewed their measurements. The measurements included height and weight as well as the widths and circumferences of the chest (breast/bust), waist, hips, biceps, thigh, and calf. These measurements were used to create baseline models for the app using the MakeHuman software (MakeHuman) [18].

### Development of the Monitor Your Avatar App Avatars

#### Overview

**Figure 1:**
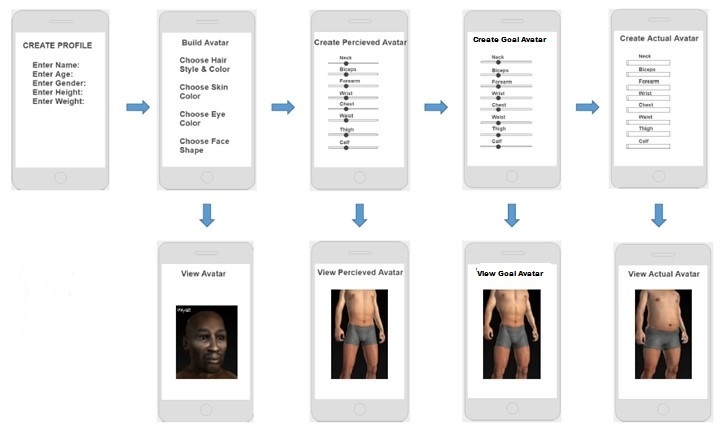
User Interaction Screens for MYA App.

The purpose of the development phase was to use the classified models and build a pipeline to display and manipulate them via a Web app. The Web app called MYA provides an interface (see Figure 1) for the participants to engage with the following avatars: *Perceived Avatar* (what adolescents think they look like), *Target Avatar* (what adolescents want to look like), and the *Actual Avatar* (what adolescents actually look like based on body measurements).

#### Procedure for Prototype Development

The prototype development consisted of three steps: (1) Modeling: generating the baseline models using MakeHuman [18]; (2) Display: rendering the models via a Web interface written in JavaScript and WebGL [19]; and (3) Analysis: saving the user-generated avatar content to a secure database for research and analysis.

#### Modeling

With the provided measurements, the models were created by setting up the gender, height, and age in MakeHuman [18]. The scanned model and measurements were used as a reference to match the body type and create the baseline model that is geometrically similar to the scanned model. MakeHuman models are not fragmented into body parts and manipulating one area had no effect on the other. Since we were seeking local control and manipulation based on measurements of specific body parts, we had to use the Blender 3D software (Blender) [20] to fragment the model into body parts. A new model whose individual body parts could be manipulated by varying the measurements using sliders was created. With this model, the slider could be used to manipulate each body part independently and provide the ability to export the functionality to the Web.

#### Display

The models from the Blender software were exported as a single JSON file [21] that contained the location and name of all the baseline models. Once a representative sample was selected based on height, weight, and gender input we used Three.js [22], a Web-based, 3D-rendering application programming interface, for loading and displaying the appropriate baseline model with the manipulating sliders. The manipulation of the body parts is done independently by an algorithm that morphs the 3D object based on predetermined minimum and maximum 3D surfaces for each body part. The *Actual Avatar* is generated by having the users input the body measurements and algorithmically calculate the morph targets for this input set.

#### Analysis

When the user completed the three avatars, the measurements of all body parts for all the avatars were retrieved and the WebGL content was saved into an image format. The measurements of body parts were saved onto a spreadsheet. All three avatars were compiled onto a screen capture and the final state of the app was saved. A server-side script was written to give administrative access to the research team to easily download this data for all the adolescents.

### Prototype Testing of the Monitor Your Avatar App

#### Overview

The purpose of the third phase was to determine the acceptability and usability of the MYA app in male and female adolescents aged 15-18 years. Acceptability was defined as thoughts on viewing the avatars and visual representation, as well as comfort level of using, intent to use, and satisfaction with the app. Usability was defined as ease of using and actual use the app, need to look for help when using the app, understanding of how to use the app, and engagement of the app.

#### Procedure for Prototype Testing

Participant inclusion criteria included the following: (1) in grades 9-12; (2) able to speak, read, and write English; (3) has not been diagnosed with an eating disorder or depression, as provided by a school nurse; and (4) be comfortable wearing form-fitting clothes such as compression shorts, leggings, form-fitting tank top, or swimwear for taking measurements. These adolescents were not part of the body scan study completed for the classification phase. The research team introduced the study to 45 adolescents in two high school physical education classes. One adolescent elected not to be part of the study and two others were absent on the day of data collection.

During data collection, the adolescents wore form-fitting clothes. The research team measured the height of the adolescents using a stadiometer and their weight and body fat percentage using a Tanita body composition analyzer. We also measured the adolescents’ body parts—biceps, chest/bust, waist, hips, thigh, and calf—using a body tape measure to obtain circumferences. The girls also had their bust girth measured, in addition to their chest/bust, for entry into the *Actual Avatar* feature.

**Figure 2:**
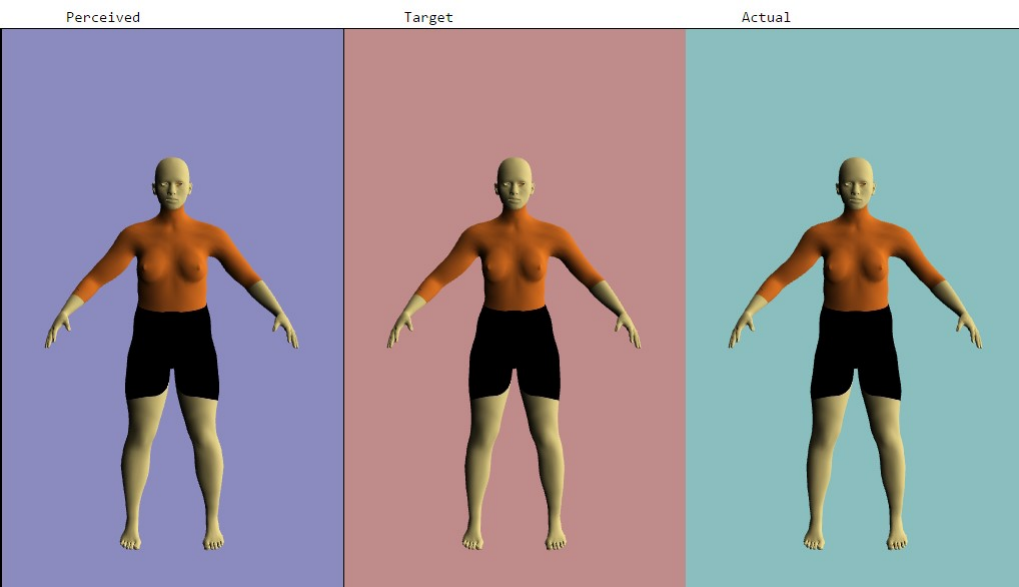
Example of three female avatars (front view).

**Figure 3:**
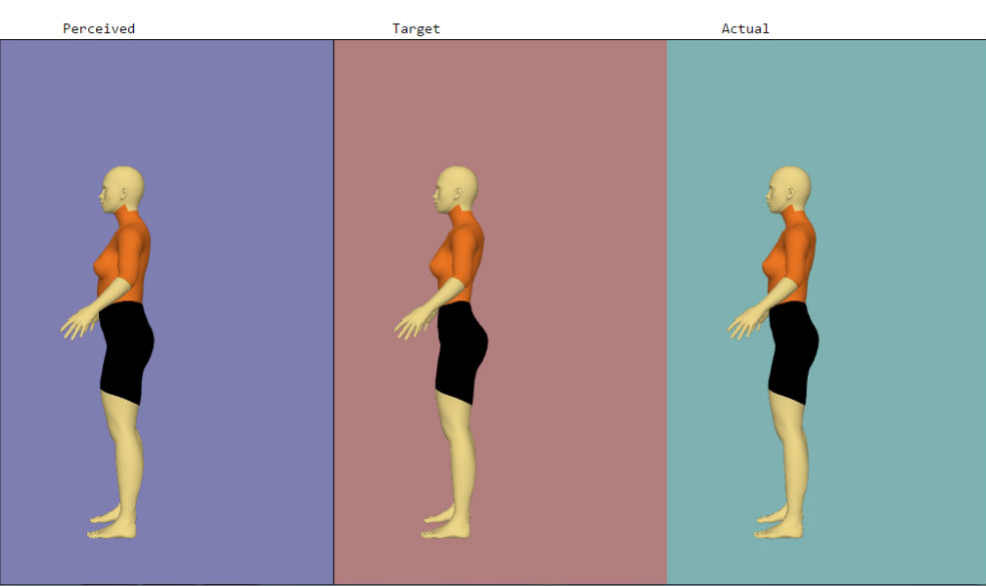
Example of three female avatars (side view).

After measurement, the adolescents entered an assigned identification number, their gender, measured weight, and measured height into the app to populate a baseline avatar using the computer located in the school library. They then designed the avatar to represent how they currently perceive their bodies to look (*Perceived Avatar*). The app allowed each adolescent to make the body parts (ie, biceps, chest/bust, bust girth, waist, hips, thighs, and calves) bigger or smaller using the slider. Their completed *Perceived Avatar* then generated on the same screen so they could design it to represent how they wanted their bodies to look within realistic, healthy parameters (*Target Avatar*). This feature was incorporated so the adolescents could work from their current perception rather than having to start over. On the same screen and to the right of the *Perceived* and *Target Avatars*, the adolescents generated an *Actual Avatar* by entering their body part measurements into the app. Figure 2 displays the three avatars after completion. The avatars could be rotated 360 degrees. Figure 3 displays the three avatars from the side view.

The adolescents were then asked to answer questions about their reactions to creating and viewing the avatars to assess acceptability and to complete the Software Usability Survey. The open-ended reaction questions posed to the adolescents included the following: “What do you think about being able to view avatars of yourself?” “Do you think the avatars are a good representation of yourself? Explain why or why not.” “How comfortable were you creating and viewing your avatars?” “In the future, would you use these avatars to see how your body parts are changing? Explain why or why not.” “What do you like about the avatars?” “What do you not like about the avatars?” “What would you change about the avatars?”

The Software Usability Survey is a short usability 7-point Likert-type scale survey informed by the product and best practices in software engineering [23,24]. The items used to identify usability for the MYA app included the following: (1) simplicity of navigation from one page to another, (2) ease of control to view the avatars and their body parts, (3) ease to manipulate each body part and make it look more muscular, (4) need to look for help when working with the app, (5) understanding of what the three avatars are and how to view and manipulate them, (6) ability to keep the user engaged and not quit, and (7) overall satisfaction with ease of using the app.

#### Analysis

SPSS version 23 (IBM Corp) was used to conduct the descriptive statistics of the sample. The adolescents’ reactions to the MYA app were analyzed using content analysis [25]. The content analysis process included three phases: preparation, organizing, and reporting. The preparation phase included selecting the unit of analysis and classifying the data as a whole. The organizing phase included developing a categorization matrix and coding the data per the categories. The reporting phase included the results of the analyzing process and the results. The frequencies of the Software Usability Survey were analyzed and calculated into percentages.

## Results

The sample characteristics for prototype testing are highlighted in Table 1, with the majority of the adolescents being female (28/42, 67%), age 16 years (16/42, 38%), white (35/42, 83%), non-Hispanic (36/42, 86%), in grade 10 (20/42, 48%), healthy weight for females (23/28, 82%), and obese for males (7/14, 50%).

**Table 1:** Sample characteristics of the adolescents (N=42).

Sample characteristics		n (%)
**Age in years**		
	15	12 (29)
	16	16 (38)
	17	11 (26)
	18	3 (7)
**Gender**		
	Female	28 (67)
	Male	14 (33)
**Race**		
	White	35 (83)
	More than one race	2 (5)
	Asian	2 (5)
	Black American Indian	2 (5)
	American Indian	1 (2)
**Ethnicity**		
	Hispanic	6 (14)
	Non-Hispanic	36 (86)
**Grade level**		
	9	2 (5)
	10	20 (47)
	11	16 (38)
	12	4 (10)
**Female BMI^a^** **-for-age category^b^**		
	Underweight	0 (0)
	Healthy weight	23 (82)
	Overweight	4 (14)
	Obese	1 (4)
**Male BMI-for-age category^b^**		
	Underweight	0 (0)
	Healthy weight	4 (29)
	Overweight	3 (21)
	Obese	7 (50)

^a^BMI: body mass index.

^b^Underweight: less than the 5th percentile; healthy weight: 5th percentile up to the 85th percentile; overweight: 85th to less than the 95th percentile; obese: equal to or greater than the 95th percentile.

To determine acceptability, the adolescents’ responses to the reaction questions (see Table 2) were analyzed using content analysis [25]. When asked what the adolescents thought about being able to view the avatars of themselves, 37 out of 42 adolescents (88%) provided positive feedback. For example, one adolescent commented about the actual and target representations: “I think it's good to see what you actually look like, then being able to see what your personal target is” (healthy-weight, 15-year-old female).

Another adolescent compared the app to the mirror: “It's nice to see what I actually look like opposed to looking in the mirror” (obese, 16-year-old male). When asked if the avatars were a good representation of themselves, 38 out 42 (90%) responded “yes.” One adolescent explained the following: “Yes, because it shows what my body looks like and what I think it looks like” (healthy-weight, 16-year-old female).

Another adolescent indicated how this was something new: “Yes, I've never done something like this. I think it is a good representation” (overweight, 17-year-old male). One adolescent did not think it was a good representation: “Not really, because I think more measurements should be taken” (obese, 18-year-old male).

When asked if they were comfortable viewing the avatars, 41 out of 42 (98%) indicated they were comfortable. The research team categorized the level of comfort into three categories: very comfortable, somewhat comfortable, and comfortable based on the explanations the adolescents provided (see Table 2). Almost half of the adolescents (18/42, 43%) were very comfortable creating and viewing their avatars. When asked if the adolescents would use the avatars in the future to see how their bodies changed, 41 out of 42 (98%) responded “yes” (see Table 2).

The adolescents explained the reasons they would use the app in the future. Most of the adolescents indicated they would use the app to track progress and goals (15/42, 36%) and to see the changes or differences in their body parts (11/42, 26%). The following are examples of why the adolescents would use the avatars in the future:

Yes, it would be very helpful because sometimes your progress can't be seen on the body but is realized in measurements or on other body/perspectives.

Healthy-weight, 16-year-old female

*Yes, because visual representation would help build confidence in achieving the goal*. [Overweight, 16-year-old female]

Yes, I would like to see the pictures of my before and after results.

Obese, 16-year-old male

There were several reasons the adolescents liked the avatars. Almost half of the adolescents (18/42, 43%) indicated they liked how the avatars were realistic, while almost a third (15/42, 29%) liked how they could move the avatars to see them from different angles (see Table 2). Adolescents commented on what they liked:

*That they are suited for the gender that you are and body type*. [Healthy-weight, 17-year-old female].

*I like how they're realistic*. [Healthy-weight, 15-year-old female].

*How you can play around with them and see what you look like with certain numbers*. [Obese, 16-year-old male].

When asked what the adolescents did not like about the avatars, approximately one-third (13/42, 31%) indicated there were not enough details or customization options with the avatars, while 13 out of 42 (31%) commented there was nothing to dislike.

*They do not show definition of muscle.* (Obese, 16-year-old male).

*They don't have as much detail, with looks and more measurements.* (Healthy-weight, 16-year-old female).

The suggestions the adolescents made when asked about what they would change about the avatars included the appearance of the avatars, such as the clothing, hair, etc (16/18, 38%). One participant stated, “I think I would change the avatars to look more like the person making the avatar so they can really feel more into it and help view the different avatars of the person” (healthy-weight, 16-year-old female).

**Table 2:** Responses to the questions identified by the adolescents (N=42).

Questions and answers		n (%)
**What do you think about being able to view avatars of yourself?**		
	Positive feedback	37 (88)
	Neutral feedback	4 (10)
	Negative feedback	1 (2)
**Do you think the avatars are a good representation of yourself?**		
	Yes	38 (90)
	No	4 (10)
**How comfortable were you creating and viewing your avatars?**		
	Very comfortable	18 (43)
	Comfortable	15 (36)
	Somewhat comfortable	8 (19)
	Neutral	1 (2)
**In the future, would you use these avatars to see how your body parts are changing?**		
	Yes	41 (98)
	No	1 (2)
**Reasons the teenagers would use the app in the future**		
	To track progress/goals	15 (36)
	To see the changes/differences in body parts	11 (26)
	Helpful to see visual representations	8 (19)
	Accurate representations	4 (10)
	It was fun/interesting	2 (5)
	Yes, with no explanation	1 (2)
	No	1 (2)
**What do you like about the avatars?**		
	Exact/actually look like/realistic	18 (43)
	Ability to move them/3D	12 (29)
	Ability to compare avatars	4 (10)
	It was fun/cool	3 (7)
	Others’ perspective	2 (5)
	App is anonymous	1 (2)
	Target Avatar	1 (2)
	No response	1 (2)
**What do you not like about the avatars?**		
	Needs more details/customization	13 (31)
	Nothing	13 (31)
	Appearance of avatars/clothes	8 (19)
	Difficulty seeing body changes made	3 (7)
	Not accurate	2 (5)
	Uncomfortable to see self	2 (5)
	Difficulty moving avatars	1 (2)
**What would you change about the avatars?**		
	Appearance of avatars/clothing	16 (38)
	Nothing	9 (22)
	Add more details	8 (19)
	Able to see body changes better	7 (17)
	Able to move avatars better	1 (2)
	Add diet to the app	1 (2)

The results of the 7-point Software Usability Survey indicated that the adolescents found the app simple to navigate (rating of 6 or 7; 40/42, 95%), found it easy to control the viewing of the avatars (rating of 6 or 7; 31/42, 74%), and found it easy to manipulate each body part and make it look muscular (rating of 6 or 7; 24/42, 57%). The adolescents had the need to look for help several times (rating of 1 or 2; 42/42, 100%), had a better understanding of what the three avatars are and how to view them (rating of 6 or 7; 40/42, 95%), were engaged by the app (rating of 6 or 7; 41/42, 98%), and experienced overall satisfaction with the ease of the app (rating of 6 or 7; 41/42, 98%).

## Discussion

### Principal Findings

The purpose of the prototype testing was to assess the acceptability and usability of an avatar-based, theoretically derived mobile app, the MYA app. Prior to the app being ready for prototype testing, two phases were completed that consisted of classification and development. Once completed, the app prototype was tested with a sample of male and female high school adolescents. The adolescents created and viewed the three avatars: *Perceived Avatar*, *Target Avatar*, and *Actual Avatar*. The adolescents had positive reactions to the avatar app and being able to view avatars that represented them. Almost all of the adolescents indicated some level of comfort viewing the avatars and would use the app in the future to see how their bodies change over time.

Adolescents who evaluated a Web-based substance abuse intervention also indicated high ratings for overall usability features such as ease of use and future use [26]. The ease of manipulation of the body parts and making the avatars look more muscular resulted in a lower-percentage rating from the adolescents than the other usability survey items. The responses regarding the viewing of the avatars support this lower score. The adolescents indicated that more details and customization of the avatar bodies were needed and that it was difficult to see the changes made to the avatars. These responses may explain the lower ease of manipulation score. In further adaptations, there is a need for the avatar bodies to be more detailed with an easier view of the body changes from one avatar to the next.

The adolescents indicated they would use the avatars in the MYA app in the future to monitor their body shape changes. This is similar to participants who were assigned a virtual representation of their physical selves, an unchanging virtual representation, or no virtual representation. Those who witnessed the virtual representation of their physical selves engaged in more voluntary physical activity than those who saw an unchanging virtual self or no virtual representation [8].

Ridgers and colleagues [27] conducted a systematic review of the effectiveness of youth wearable tracker devices to increase physical activity levels among children and adolescents. They found these devices have the potential to increase activity levels as the adolescents self-monitor their progress and set their goals. The MYA app is similar in that it can be used to provide feedback of their body shape progress and monitor their goals. Ridgers and colleagues [27] recommended that research be conducted to establish how youth engage with technology over longer periods of time. The adolescents indicated future use of the MYA app; however, more research is needed to examine their engagement with the app over time.

### Limitations

This study is not without limitations. Due to the sample of adolescents being relatively small and nondiverse, there was not enough power to detect group differences. Another limitation is that the adolescents only tested the app at one session. To understand the acceptability and usability over time, it is necessary for the adolescents to interact with the app over a longer period of time with several recurrent uses. The app did not have this capability at the time of testing.

### Implications

This app allows teens to have visual representations of their bodies—perceived, target, and actual avatars— interactive individualized programs, access to immediate feedback, and a method to monitor changes to their bodies as a result of positive changes in health behaviors. This visual representation is different from what adolescents currently have access to, such as digital scales and BMI calculators. Adolescents continue to be overweight and obese while physical activity decreases as they get older. Adolescents who identify with and use these avatars who represent them can monitor and reach their body shape and health goals. While this app may be user driven, it also has the capability for health care providers, parents, and adolescents to have a visual communication tool to discuss adolescents’ body perceptions and goals.

### Conclusions

Avatar-based mobile apps, such as the MYA app, provide immediate feedback and allow users to engage with images that are personalized to represent their perceptions and actual body images. Adolescents appreciate the use of weight-related messages that are personalized for them [28]. The high school students found the avatars of the MYA app easy to use, were comfortable viewing their avatars, and would use the avatars in the future to monitor how their body changes. Overall, they were satisfied with the MYA app; however, they made suggestions on how to make the app more appealing to high school males and females. This pilot study adds to the increasing but limited research of using games to improve health outcomes among high school adolescents. There is a need to further adapt the MYA app and gather feedback from a larger number of high school adolescents, including those from disadvantaged backgrounds, while testing adherence to the app over time.

## Abbreviations

BMI: body mass index
MYA: Monitor Your Avatar
